# A Survey on the Roadmap to Mandate on Board Connectivity and Enable V2V-Based Vehicular Sensor Networks

**DOI:** 10.3390/s18072207

**Published:** 2018-07-09

**Authors:** Barbara M. Masini, Alessandro Bazzi, Alberto Zanella

**Affiliations:** National Research Council of Italy (CNR), Institute of Electronics, Computer and Telecommunication Engineering (IEIIT), v.le Risorgimento, 2, 40136 Bologna, Italy; alessandro.bazzi@ieiit.cnr.it (A.B.); alberto.zanella@ieiit.cnr.it (A.Z.)

**Keywords:** connected vehicles, vehicular sensor networks, IEEE 802.11p, cellular-V2X, LTE-V2V, VLC, mmWave, 5G, performance comparison

## Abstract

Vehicles will soon be connected and will be interacting directly with each other and with the road infrastructure, bringing substantial benefits in terms of safety and traffic efficiency. The past decade has seen the development of different wireless access technologies for vehicle-to-everything (V2X) communications and an extensive set of related use cases have been drafted, each with its own requirements. In this paper, focusing on short-range communications, we analyze the technical and economic motivations that are driving the development of new road users’ connectivity, discussing the international intentions to mandate on board devices for V2X communication. We also go in depth with the enabling wireless access technologies, from IEEE 802.11p to short-range Cellular-V2X and other complementary technologies, such as visible light communication (VLC) and millimeterWaves, up to hybrid communication and 5G. We conclude our survey with some performance comparison in urban realistic scenarios, underlying that the choice of the future enabling technology is not so easy to predict and mostly depends on mandatory laws at the international level.

## 1. Introduction

In the near future, vehicles will be connected, becoming mobile sensors of traffic, pollution and driver behaviors. A car today is, in fact, already equipped with thousands of sensors collecting information about the vehicle and its surroundings [1]. When wireless connectivity will also be available, the data collected on board could be forwarded to other vehicles in the surrounding area, road side infrastructures and remote data centers through vehicle-to-everything (V2X) communications, thus enabling new services and applications ranging from safety to traffic efficiency and infotainment. Wireless technologies can, in fact, extend the vehicle’s ability to see further down the road by providing 360-degree non line-of-sight awareness. This capability complements the use of sensors, which rely on line-of-sight and typically good weather conditions, limiting their range and capabilities under certain conditions [2].

Hence, the choice of the best wireless technology to enable vehicular connectivity is becoming a crucial issue, discussed both at the round tables of researchers, and at an industrial level; new players are entering the game, not only from the automotive and telco sectors, but also from information technology fields, such as Google, (see, e.g., the Google car), and original equipment manufacturer (OEM), such as Bosch (Stuttgart, Germany) (see, e.g., the artificial intelligence (AI) system on board future Level 3 autonomous cars) and international entities are fighting to propose the wireless access technology that they believe in more. This is justified, for example, by the OEM expected revenues from monetization of automotive sensor data that are expected to reach 706 million US$, representing a compound annual growth rate (CAGR) of 46.8% according to ABI Research a market-foresight advisory firm providing strategic guidance on the most compelling transformative technologies; it foresees that cars contributing sensor data to ingestion platforms is expected to grow to over 60 million by 2023 [3] .

About the choice of the enabling technologies, the Car-to-Car Consortium, for example, is leaning toward the IEEE 802.11p and its related standards (such as intelligent transportation systems (ITS)-G5), since it represents the most tested and consolidated technology for vehicular communications and have shown good performance also in challenging scenarios [4]. The 5GAA is instead promoting C-V2X, which includes both long- and short-range communications based on the cellular standards; in their opinion, short-range C-V2X has the advantage of being included in the same chipset as long-range communications, also promising better performance than IEEE 802.11p due to more advanced solutions at the physical and medium access layers and taking advantage of a more solid industrial ecosystem to guide its future evolution [5]. Besides these two main technologies, other complementary wireless access technologies are under investigation, such as visible light communication (VLC) and mmWave [6], in order to improve security, reliability and/or latency in specific scenarios.

The objective of this paper is to provide an insight of the challenges posed by short-range vehicle-to-vehicle (V2V)-based vehicular sensor networks (VSNs) starting from economic motivations up to requirements and related enabling technologies, also presenting an overview of the potential mandatory rules that are being discussed at an international level to integrate connectivity on board of vehicles. Differently from other surveys already published in the literature, in this paper: (i) we narrow the attention to short-range V2V-based applications, (ii) we discuss the design choices and factors that determine the development of different wireless enabling technologies, (iii) we discuss potential economic motivations to install wireless technologies on board and (iv) we provide an overview of how international organizations and countries are moving to provide suitable business models and potential mandatory rules to push connectivity on board and thus efficiently support manufacturers to develop applications that employ V2V communications. Besides touching these main three points, we also provide a description of the main enabling technologies, from IEEE 802.11p and its related standards, to Cellular-V2X (C-V2X) and other complementary technologies, such as VLC and mmWave, opening to future vehicular networks.

The paper is organized as follows: in Section 2, the main related works are examined, highlighting the main differences with our proposal; in Section 3, the economic pushes to vehicular communications are discussed; then, the main requirements and applications are highlighted. In Section 4, wireless enabling technologies are detailed with a glance at 5G and hybrid systems, in Section 5, mandatory rules at international levels are presented, and, in Section 6, example results of performance comparison are provided. Finally, in Section 7, our conclusions are drawn while opening up to future perspectives.

## 2. Related Works

We address vehicular sensing applications, where vehicles cooperate to sense and collect environmental data that can be requested, for example, by public administrations or private data centers [7]. A general vision on architectures and technologies for V2X can be found in [8].

Narrowing, indeed, the field of research to VSNs, some principles and challenges were provided in [9,10], describing typical topology and architecture and exploring the main requirements at the different levels of the protocol pillar. In [11], a survey of the most significant approaches for wireless and mobility modeling in vehicular networks’ simulation is given, listing the main channel models that have been employed in the literature for IEEE 802.11p and related standards. In [12], an overview on the communication and networking aspects of vehicular ad hoc networks is presented, but the paper dates 2008 when the only standard for these kinds of networks was IEEE 802.11p. A brief report on vehicular traffic flow models and some radio channel basics required for realistic assessments of systems and protocols are also discussed. A survey on mobility models in VSNs is proposed in [13], also providing methodologies for the cooperation between mobility models and network simulators.

An extended survey, but related to vehicular social networks, has been provided in [14], where social characteristics and human behavior are considered for their impact on vehicular networks and the impact of social-based protocols is taken into account to identify socially-similar nodes sharing common interests and roads. In [15], VSNs operating in a smart city are discussed, followed by an overview of a range of associated technologies. The authors also highlight the importance of an accurate topological structure and propose an information source selection model to improve the attainable network capacity. In [16], an analysis of the strengths and uses of dedicated short-range communications (DSRC) and cellular vehicular networks is presented, exploring strategies, beyond government mandates, to encourage vehicular network deployment.

Several works related to VSNs focus also on data traffic mapping and data analytics. In [17], for example, VSNs are considered for traffic monitoring in urban areas: probe vehicles, such as taxis, buses and floating cars in the city of Shangai are used as mobile sensors of the urban traffic and send the reports to a traffic-monitoring center for traffic estimation. The authors of this work propose a method to fully exploit the acquired data and also estimate the traffic condition of the unsampled roads.

Differently from the cited references, in this paper, we aim at the underlying motivation to VSNs also from an economic perspective; then, we focus on actual and future wireless access technologies, also presenting the roadmap for potential mandatory rules on vehicular connectivity at international level and we conclude with a performance comparison in realistic scenarios of the two main candidates technologies for short-range VSNs: IEEE 802.11p and LTE-V2V.

## 3. Economic Motivations, Applications and Requirements for VSNs

Why are VSNs so hot? V2V communication in conjunction with automated vehicle technologies could provide a higher level of predictability than sensors, radar and cameras alone. V2V allows vehicles to share their intention (e.g., sudden lane changes) and exchange sensor data. The sharing of these types of information will help vehicles better anticipate and react to the movements of other nearby vehicles [2].

The importance of connected vehicles is also demonstrated by the fact that, for the first time, the European Commission (EU) adopted in November 2016 a European strategy on cooperative-intelligent transportation systems (C-ITS), to deploy vehicles that can “talk” to each other and to the transport infrastructure on EU roads as of 2019 [18], and, in December 2016, the National Highway Traffic Safety Administration (NHTSA), announced the proposal of an introduction of rules to mandate V2V communication on light vehicles from 2019 (as it will be better detailed in Section 5).

Studies have estimated the market potential of cooperative, connected and automated driving to be worth dozens of billions of euro annually and the creation of jobs could run into the hundreds of thousands [18]. Connectivity, in fact, will provide important applications, which translates into new business model opportunities for municipalities, companies, and insurance, as well as road and connectivity infrastructure owners.

**Figure 1:**
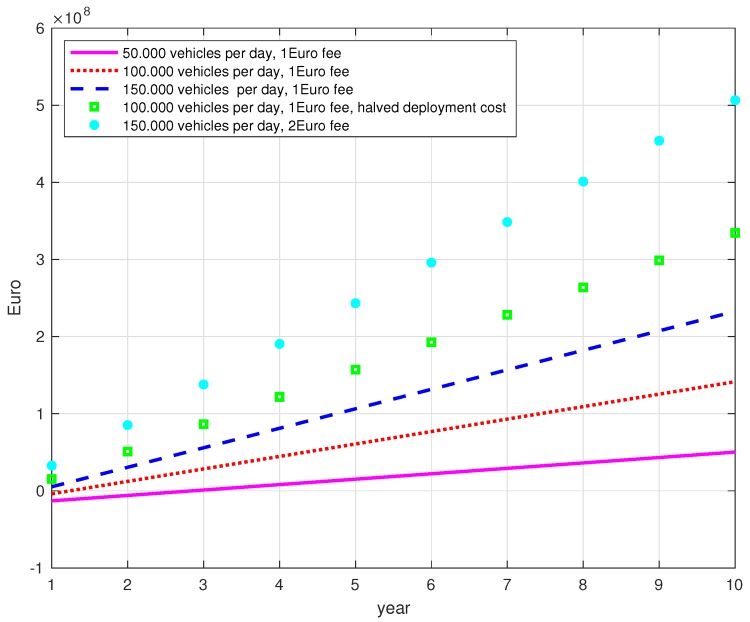
Achieved profit in Euro for different scenarios in a 10-year projection.

A first insight into the deployment costs for V2X and revenue analysis for financially and socially beneficial commercialization are provided in [19], which represents the first outcome of the 5G-PPP Automotive working group. In [19], the authors provide an estimation of investment cost and profit for a 10-year horizon considering a 100 km highway segment with a traffic density of 100,000 vehicles/100 km per day, a 10% yearly penetration rate, a service fee of 1 Euro per vehicle with an income of 50% of the fee and a deployment cost made by the sum of raw capital expenditure (CAPEX) and operating expenditure (OPEX) considerations; they showed that it would be necessary five years after the initial investment to see some profit, but a profit of almost 70 MEuro can be expected in 10 years. Following similar considerations, in Figure 1, we plotted the achieved profit for different scenarios varying the traffic density and the service fee in 100 km highway and 100% penetration rate. We assume, as in [19], an income of 50% of the fee. As a raw estimation of deployment costs, we assume a CAPEX equal to 20 MEuro every 100 km and OPEX of 2.1 MEuro per year. It can be observed that the profit is negative only for the first year of the less dense scenarios with 50,000 or 100,000 vehicles with 1 Euro service fee. In five years, for example, and a density of 150,000 vehicles per day, the profit is over 100 MEuro. Of course, these are only qualitative estimations, but it is not difficult to think that, with a good business case, they may be helped by some mandatory rules, and the investment would provide high incomes.

In Table 1, some example requirements of the main typical applications are reported. Table 1 refers to V2V applications, enabled by both the periodic exchange of beacon messages and event driven messages. We did not report in this paper applications based on vehicle-to-infrastructure (V2I); for requirements related not only to V2V applications, the reader may refer, for example, to [20]. From Table 1, it could be observed that the most stringent requirements are on beacon frequency (typically 10 Hz) and latency (typically less than 100 ms). In most of the applications, the throughput per link is not very stringent, although issues arise when the number of vehicles becomes high. It must also be underlined that all the application requirements reported require a reliability higher than 90%; in particular, for cooperative maneuvers, such as merging traffic turn assistance or lane changing, the reliability should be ≥99%. Positioning represents another important aspect, since actual GPS accuracy could not be sufficient when automated connected vehicles will be on the road. In fact, from Table 1, it can be observed that typical applications require a position accuracy of about 30 cm, whereas the actual GPS system allows an accuracy around 7 m (1–1.5 m of accuracy in the best cases) [21].

**Figure 2:**
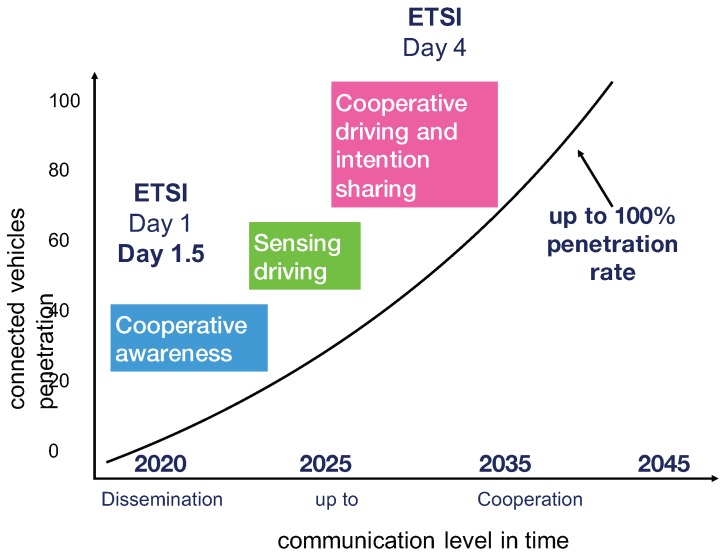
Applications roadmap of penetration of connected vehicles and ETSI priorities.

Through the C-ITS Platform, the EU also identified a list of services as candidates for early deployment. The so-called Day 1 services are considered fully mature and relevant for realizing the societal benefits in terms of road safety and reduced emissions. Day 1.5 services follow closely, up to longer term Day 4 services [18]. In Day 1 services, we can summarize applications such as hazardous location notifications, emergency brake light, probe vehicle data, whereas in Day 1.5, connected and cooperative navigation, vulnerable road user protection, etc. In Figure 2, three main steps provided by an always higher level of communication and penetration are sketched up to Day 4 services.

## 4. Wireless Enabling Technologies

More than one communication technology can be considered today for V2V connectivity. In this section, we report the actual candidates and their potential interoperability and complementarity.

### 4.1. IEEE 802.11p and Related Standard

Standardized by the IEEE in 2009 and included in the overall IEEE 802.11 standard in 2012, IEEE 802.11p (and its related standards, such as ITS-G5 in Europe) has been referred to as the standard for vehicular communication until 2017. Since it is ready and tested, it does not require fine synchronization, and it is based on a fully distributed medium access protocol, well known and fairly reliable. In addition, products are available and can be installed on board.

The 70 MHz band is subdivided in seven channels, each one of 10 MHz: one channel is used as the control channel (CCH), whereas the remaining channels are for service (SCH) [22,23]. In Table 2, we show the channel allocation in Europe for ITS G5 and in the US for IEEE 802.11p with also the maximum effective isotropic radiated power (EIRP) on the corresponding channels.

IEEE 802.11p is based on 64 orthogonal frequency division multiplexing (OFDM) subcarriers of which 52 are used for transmission (48 data subcarriers and four pilots). Eight possible combinations of modulation and coding scheme (MCS) are provided; depending on the adopted MCS, the raw data rate varies between 3 and 27 Mb/s. The fast exchange of information is made possible by the IEEE 802.11p medium access control (MAC) level, which enables very efficient communication-group setup without much of the overhead typically needed in nomadic IEEE 802.11a/g Wi-Fi networks, simplifying the basic service set (BSS) operations in a truly ad hoc manner for vehicular usage through the so-called outside of context of BSS (OCB) mode, allowing communication between users without requiring authentication/association between them or with an access point [23].

At the MAC layer, IEEE 802.11p adopts carrier sensing multiple access with collision avoidance (CSMA/CA). On the one hand, it allows a fully distributed and uncoordinated access to the wireless channel, with no need for a resource allocation procedure. On the other hand, however, it implies a not negligible resource waste due to frequent collisions as the channel use increases under heavy traffic conditions, impacting reliability. Regarding VSNs-based applications, the MAC layer does not offer any mechanism for reliable broadcasting, and can suffer from poor performance at a high traffic load, which will be the normal operating condition when the connected vehicles penetrate the market.

### 4.2. Cellular-V2V (C-V2V)

Despite much effort in the last 15 years and several projects with hundreds to thousands of vehicles, no connected vehicles based on IEEE 802.11p are on the field. In the meantime, the world of mobile communications has sprinted forward and C-V2X is now referred to as one future reference standard for connected vehicles. Inside the general world of C-V2X, short-range LTE-V2X represented the first step in this direction. Standardized in June 2017, LTE-V2X exploits the existing infrastructure/system and sketches out the road to 5G vehicular networks. The first step in this direction was made by LTE-Direct.

“You can think of LTE-Direct as a sixth sense that is always aware of the environment around you”, said few years ago Mahesh Makhijani, technical marketing director at Qualcomm. LTE-Direct represented the first modification to the most wide diffuse communication technology, the cellular one. The massive growth in the number of connected devices and in traffic volume and the increasingly wide range of applications with varying requirements and characteristics pushed long term evolution (LTE) to face new challenges targeting device to device (D2D) communication. Hence, for the first time in history, a cellular system allows direct communication between peers. LTE-Direct is supported by the 3GPP LTE Advanced Release 12 and provides D2D communications over cellular networks. It exploits the LTE infrastructure for resource allocation, timing, and authentication; then, it allows LTE-Direct based devices to communicate directly with one another when in proximity [24].

However, it is only with 3GPP Release 14, frozen in June 2017, that the support of short-range V2X communication was added to LTE [25,26]. The new specifications define modifications to D2D: direct V2V communication adopts the uplink setup of LTE at the physical (PHY) and MAC layers, thus single carrier-frequency division multiple access (SC-FDMA) is applied. In the frequency domain, the channel bandwidth is subdivided into groups of orthogonal subcarriers equally spaced by 15 kHz while, in the time domain, the signal is split into frames of 10 ms, which are in turn made of 10 subframes of 1 ms. The subframe is additionally discretized in two time slots of 0.5 ms. Considering the time-frequency matrix of resources, one time slot and 12 contiguous subcarriers constitute a resource block (RB). The Sidelink interface has been tuned to counteract the severe Doppler experienced at high speed: out of the total 14 OFDM symbols carried by a subframe, the last is left unused to allow timing adjustment and Tx/Rx switch and 4 are dedicated to channel estimation, thus leaving only nine data symbols. Advanced coding techniques and an almost continuous variation of 21 MCS combinations are adopted which contribute, together with the possible use of portions of the bandwidth, to a higher reliability and range with respect to IEEE 802.11p.

Release 14 also introduces two new functioning modes (Mode 3 and Mode 4) to solve the issue of resource allocation for V2V communications [27,28]: in Mode 3, two vehicles directly communicate, but the reservation and scheduling of the resources is performed by the cellular infrastructure; in Mode 4, vehicles autonomously manage also the resources adopting a fully distributed procedure. Hence, in sidelink Mode 4, there is no control of the evolved NodeB (eNB) on direct links, as V2V public safety applications should also work in the absence of network coverage. The resource selection procedure is performed autonomously by vehicles that typically have to sense the medium before transmitting with semi-persistent scheduling (SPS); this means that the algorithm applies real-time measurements of the received power, with the aim of keeping a tolerable level of interference, while increasing the reuse of resources.

### 4.3. Visible Light Communication (VLC)

VLC allows high data rate transmission at the same time as illumination, by exploiting the same main principles of Bell’s photophone transmissions dated more than 100 years ago. It is, in fact, sufficient to quickly switch on and off the light emitting diode (LED) to transmit a wide bandwidth signal invisible to the human eye and completely safe for the human body, by exploiting the wide and free optical spectrum. VLC is, thus, naturally suited to broadcast messages. Vehicles are already equipped with LED lights that could be easily exploited for V2V communications without interfering with electromagnetic devices and allowing a high reuse factors and lower interference from neighbors thanks to the limited penetration capabilities of light [6]. Besides these advantages, it has to be remarked that VLC is susceptible to ambient light and weather conditions, covers limited distances and does not cross obstacles [29,30].

The increasing interest in this kind of communication is also demonstrated by the development of the IEEE 802.15.7 standard, which defines the PHY and MAC layers for the visible light spectrum and allows for supporting audio and video services also in mobile visible links [31]. At the PHY layer, three are the available operating modes which differ in the adopted modulation and coding scheme [31,32], allowing data rate from 11.67 to 96 Mb/s. At the MAC layer, the IEEE 802.15.7 standard foresees the use of CSMA/CA: each node transmits only if the medium is sensed as idle after a random backoff interval time; then, the message is considered correctly received only upon the reception of an acknowledgment at the transmitter. A revision of the standard, called IEEE 802.15.7r1, is foreseen to better address vehicular communications as a VLC use case. In this case, the standard considers the requirements of vehicular communications and aims to enhance mobility, data rates, robustness and the networking protocols [33].

### 4.4. MmWave Communications

MmWave communication is a promising technology for also providing a very high data rate for mobile devices. MmWave works with wavelength on the order of millimeters and includes the frequency band [30 300] GHz [34].

As for VLC, the propagation loss is much higher than in traditional radio frequency bands. Specifically, in this case, the free space propagation loss is proportional to the square of the carrier frequency. With a wavelength of about 5 mm, for example, the free space propagation loss at 60 GHz is 28 dB higher than at 2.4 GHz [35]. In addition, the oxygen absorption in the 60 GHz band has a peak, ranging from 15 to 30 dB/km. Hence, transmissions can easily be blocked by obstacles. This aspect implies on the one hand that signals could be limited in roads, but, on the other hand, the system has limited coverage and reliability.

In spite the challenging issues related to the higher frequencies, mmWave communications are highly considered for future VSNs with the aim to provide a very high capacity.

More than one international organization worked on its standardization, including the European computer manufacturers association (ECMA) [36], IEEE 802.15.3 Task Group 3c (TG3c) [37], IEEE 02.11ad standardization task group [38] and consortia, such as the WirelessHD consortium, and the Wireless Gigabit Alliance (WiGig).

As far as IEEE 802.11ad is concerned, it specifies the PHY and MAC layer in the 60 GHz band. In the physical layer, two operating modes are defined: the first is based on OFDM for high performance applications and high data rate, the second is based on a single carrier (SC) for low power and low complexity implementation. At the MAC layer, a hybrid multiple access of CSMA/CA and time-division multiple access (TDMA) are adopted: CSMA/CA is more suitable for bursty traffic and reduced latency, while TDMA is more suitable to support high quality of service (QoS).

MmWave radar is widely used in vehicles for applications such as adaptive cruise control and collision avoidance [39] or for opportunistic target detection and parameter estimation [40]. MmWave communications are also considered for future 5G systems. In fact, as it will be better detailed in Section 4.5, 5G new radio (NR) allows a cellular-based system to operate in a wide range of carrier frequencies from a sub-6 GHz band to an mmWave band with appropriate handling of a multi-path delay spread and phase noise depending on the carrier frequency.

### 4.5. 5G

The roadmap of vehicular communications is marked by the evolution toward 5G, which is typically addressed as the future technology that will provide three main requirements: peak data rates faster than 10 Gbps, device density higher than 1 million per km${}^{2}$ and latency lower than 1 ms. A new radio technology, called NR, is being developed to this aim.

The three major players for 5G NR standardization are the International Telecommunication Union (ITU), 3GPP and IEEE. Candidate technologies are being evaluated by ITU Radiocommunication Standardization Sector (ITU-R) through its 5D Working Party (WP) [41].

3GPP’s 5G standards are expected to be finalized with Release 15 (Phase 1) and Release 16 (Phase 2) by September 2018 and late 2019, respectively, and it is the Release 16 that includes the draft standard for 5G V2X [42].

5G works, as LTE, in frequency bands up to 6 GHz, but new frequency are also considered at 28 GHz and 39 GHz. 3GPP is also identifying regulatory requirements of direct communications between vehicles in the spectrum beyond 6 GHz in different regions: 63–64 GHz and 76–81 GHz. Hence, 3GPP is working to manage the allocation of mmWave spectrum for 5G cellular applications and it is expected to be fixed by 2018.

As an evolution of LTE, in its first phase, 5G will be based on OFDM with subframe duration fixed to 1 ms and the frame length to 10 ms. Subcarrier spacing is 15 kHz * 2n, hence each symbol length (including cyclic prefix) of 15 kHz equals the sum of the corresponding 2n symbols of the subcarrier spacing. A slot is 7 or 14 OFDM symbols (for subcarrier spacing up to 60 kHz) and 14 OFDM symbols (for subcarrier spacing higher than 60 kHz). Future development of 5G will also include a viable option to halve the subframe duration (so far, LTE-V2X supports only a fixed subframe of 1 ms) and reduce the latency. In addition, non orthogonal multiple access (NOMA) is also being studied.

The wide channel bandwidth and multiple subcarrier spacing options enable the 5G NR system to efficiently and flexibly operate from sub-6 GHz band to mmWave band with appropriate handling of multi-path delay spread and phase noise depending on the carrier frequency.

As anticipated in Section 4.4, IEEE has recently undertaken a 5G track to enhance existing standards and to evolve new technologies for the mmWave unlicensed bands. These efforts include IEEE 802.11ad/aj/ay, IEEE 802.15.3c, ECMA-387, among the others. The standardization is expected to be completed by late 2019, ahead of the much-anticipated 2020 timeline for commercial deployment [41].

### 4.6. Hybrid V2X

Hybrid systems can enhance the overall communication performance by combining and coordinating the capabilities of different access technologies in a flexible way. Since IEEE 802.11p and LTE-V2X might not be able to guarantee the diverse performance requirements under the future autonomous driving scenarios, future V2X communications also foresee the coexistence of both the technologies.

Some discussions are also ongoing on spectrum sharing and interoperability of IEEE 802.11p and LTE-V2X at 5.9 GHz [43]. In fact, since current V2X technologies are not flexible and reliable enough to serve the different requirements of future connected and autonomous driving, hybrid V2X communications can enable the coordination of multiple communications technologies and efficiently adapt to the requirements of future driving. Especially for safety applications, cooperative principles (providing every road user an equal level of access to safety information such that all have a similar safety change) are under discussion. 5GAA, supported by Qualcomm (San Diego, CA, USA), proposed to split, in the short-term, the channels and to allocate distinct 10 MHz channels at 5875–5905 MHz to each of the two technologies, while the final configuration in the long-term will apply full sharing of all available channels across the two technologies [44]. Car to Car Consortium answered that the solution proposed by 5GAA is not possible since the 176 and 180 channels are both in use by ITS-G5 and used for different services. A split as proposed will lead to adjacent band interference and potential incorrect functioning of the ITS-G5 system [45]. The Consortium proposes to extend the currently designated spectrum from 30 MHz to 50 MHz (also incorporating the support for urban rail) and to develop 5G cellular technology (including peer to peer V2x communications) outside the 5855–5925 MHz range at such a distance that non-interference is guaranteed so that both systems can be implemented in the same vehicle.

The future landscape of vehicular communications will, however, probably see the use of both LTE-V2X and IEEE 802.11p working together, even if the final policies and functionalities for interoperability still have to be clearly defined.

## 5. International Roadmap to Mandate Connectivity on Board

Given the interesting scenario of wireless enabling technologies and the availability of some of them (such as IEEE 802.11p) for years, it is now important to understand when connectivity will be on board. To answer this question, we analyzed different discussions all around the world and here we report the main documents and decisions that governance or international entities have taken.

### 5.1. When to Mandate Connected Vehicles in the United States

On 13 December 2016, in Washington DC, the NHTSA, announced the proposal of the introduction of rules to mandate V2V communication on light vehicles, allowing cars to ‘talk’ to each other with the aim to avoid crashes. The announcement was then formalized in [46], an action called “Notice of Proposed Rulemaking (NPRM)” dated 12 January 2017 in the US Federal Registry, where the NHTSA proposes the establishment of a new Federal Motor Vehicle Safety Standard (FMVSS), to mandate V2V communications for new light vehicles and to standardize the message and format of V2V transmissions. The action put the US on a path as the first in the world to mandate V2V communication on all new light vehicles. In [46], we can read: *without a mandate to require and standardize V2V communications, the NHTSA agency believes that manufacturers will not be able to move forward in an efficient way and that a critical mass of equipped vehicles would take many years to develop, if ever. Implementation of the new standard will enable vehicle manufacturers to develop safety applications that employ V2V communications as an input, two of which are estimated to prevent hundreds of thousands of crashes and prevent over one thousand fatalities annually*. Comments on the action had to be received on or before 12 April 2017 and more than 460 comments were sent, demonstrating high interest in the issue.

Following this action, it is possible to find a press article dated 1 November 2017, discussing the fact that the White House and the Department of Transportation (DOT) decided not to pursue a final V2V mandate, but the White House declined to comment [47]. However, no official documents have ever been published about this. It is instead official that the V2V Statement Attributable to the DOT, on 8 November 2017, Washington DC, declared that *the DOT and NHTSA have not made any final decision on the proposed rulemaking concerning a V2V mandate. Any reports to the contrary are mistaken and the rule remains regarding DOT’s significant rulemaking report [48]*. At the best of our knowledge, this is the last official notice from the US. More recent discussions, dated 28 February 2018, appear in newspapers only and confirm that the proposed federal rule under review by NHTSA has encountered delays, but, if approved in 2019, it would require new vehicles sold in 2023 in the US to have V2V connectivity [49].

### 5.2. When to Mandate Connected Vehicles in Europe

In 30 November 2016, in Brussels, the European Commission adopted a European Strategy on C-ITS, a milestone initiative towards cooperative, connected and automated mobility [18]. The Strategy will make it possible to deploy vehicles that can talk to each other and to the transport infrastructure on EU roads as of 2019. The strategy aims to avoid a fragmented European market in the field of C-ITS and to create synergies between different initiatives. It addresses the most critical issues, including cyber-security, data protection and interoperability and recommends action at different levels to meet the 2019 target date. In 17 May 2018, in Brussels, the European Commission proposes a comprehensive EU approach towards connected and automated mobility, setting out a clear, forward-looking and ambitious European agenda to identify supporting actions for developing and deploying key technologies, services and infrastructure. The communication also ensures that EU legal and policy frameworks are ready to support the deployment of safe connected and automated mobility [50]. The Commission recognizes that the new market of automated and connected vehicles is expected to grow exponentially with large economic benefits, with, for instance, revenues exceeding 620 billion Euro by 2025 for the EU automotive industry. In addition, the Commission will offer support in 2018 for testing the use of 5G connectivity to enable highly automated driving functions and new mobility services with a budget totaling around 50 million Euro [50]. For what concerns V2V communications, the Commission follows a technology-neutral approach in line with the EU Strategy and has not proposed mandatory deployment of specific technologies. However, it is stated in [50] that *to enable safety-related services that require very low latency, several manufacturers have committed to also fit short-range communication devices (Wi-Fi based) on vehicles from 2019 and road operators have also started to pre-deploy roadside communication infrastructure, allowing direct interaction between vehicles or between vehicles and the road infrastructure. As of 2020, 5G connectivity infrastructure is equally expected to be an important enabler of connected and automated mobility as well as empower innovative digital ecosystems around cars*. The document remarks the expected coexistence of different radio technologies in the 5.9 GHz band.

### 5.3. When to Mandate Connected Vehicles in Asia

According to “Made In China 2025” published by the Ministry of Industry and Information Technology, they are aiming to establish the key technology for intelligent driver support as “Intelligent Connected Vehicles” by 2020, and to possess the key technology for autonomous driving by 2025 [51]. There is tight collaboration between the government and industrial circles, promoting a broad and long-term range of proposals at the stage of international standardization, such as the international organization for standardization (ISO), ITU-R, ITU-T, and 3GPP.

Connected vehicles is a key topic also in Japan, since the Strategic Innovation Promotion Program (SIP) was launched in 2014. The item concerned is also under discussion in a collaborative trilateral meeting between the United States, Europe and Japan. A large-scale verification test began in 2017 to review various forms of data exchange centered on communications and security issues.

In South Korea, several test pilots are ongoing, but still neither mandatory rules nor timelines have been fixed.

### 5.4. When to Mandate Connected Vehicles in Australia

In Australia, the Australian Communications and Media Authority (ACMA) released a consultation paper on the “Proposed regulatory measures for the introduction of cooperative intelligent transport systems in Australia” about the introduction of C-ITS in the 5.9 GHz band, mirroring the European plans and implementation and issuing a new Class License, referencing the European standard, ETSI Standard EN 302 571 under section 132 of the Radio communications Act 1992, for C-ITS transceivers in vehicles, roadside infrastructure and carried by other road users including pedestrians and cyclists. The Federal Chamber of Automotive Industries (FCAI) on 23 September 2016 strongly supported this consultation paper and explained how the introduction of more automated and connected vehicles are fundamental to improving the living standards of Australians, including reducing traffic congestion and contributing positively to reductions of vehicle emissions [52].

### 5.5. When Connected Devices Will Be on Board

From the above sections about the roadmap of mandatory rules all around the world, it is clear that there are still no fixed regulations or mandatory rules about the integration of connectivity on board and that, in general, none of the mentioned Countries specifically indicate which technology should be used (even if it seems that the US are favorable to IEEE and EU mentions 5G connectivity for the first time in [50]). Independently from the rules, it is also interesting to understand how car makers are moving. The only commercially available on board unit (OBU) and road side unit (RSU) devices are based on IEEE 802.11p standard and some car makers are considering starting installations on board.

Many carmakersare starting to introduce V2V communication in their vehicles. Toyota announced plans to begin a broad deployment of V2V and V2I communications based on IEEE 802.11p in the US market from 2021 [53]. Toyota was the first automaker to deploy V2V communications in Japan beginning in late 2015. To date, more than 100,000 Toyota and Lexus vehicles with V2V have been sold there, whereas Cadillac (General Motors group, New York, NY, USA) has sold barely one-tenth that many Cadillac CTS sedans. Among the first vendors integrating V2V on board there is also Cadillac. In its Cadillac CTS, Cadillac declared that it would use IEEE 802.11p and global positioning sensors to communicate with other vehicles on the road sending thousands of messages per second to other cars within a distance of 300 m [54]. Messages share vital information, such as traffic conditions, speed, position and direction, but the system does not share any private information or recognizable details since firewalls and encryption ensure the users’ full privacy. In June 2017, Volkswagen declared that it would enable vehicles to communicate with each other as of 2019, integrating IEEE 802.11p as well [55]. When it is launched in 2019, the system will be based on warnings and information on local traffic risks that arise at short notice. In addition, Mercedes-Benz (Stuttgart, Germany) already equip, on request, its vehicles with the Drive Kit Plus to enable V2X communication all model series. Audi AG (Ingolstadt, Germany) has completed development of integrated V2X roof antennae. The series-production ready smart antenna contains an entire V2X solution, including radio and modem, global navigation satellite system (GNSS) antenna and receiver, V2X protocol stack and security, vehicle connectivity and V2X application matching ETSI Day-1 profile [56].

In contrast, Ford (Dearborn, MI, Stati Uniti) announced plans to use C-V2X and, in partnership with Qualcomm and Datang (in Beijing, China), has been testing C-V2X devices since 2017, but since commercial devices are not still available, Ford has not yet committed a launch date [53]. Extensive testing to validate CV2X radio performance was initiated in 2017 in Ann Arbor, San Diego, Aberdeen and Shanghai. A trial with C-V2X was announced in Japan on January 12, 2018 in cooperation with Continental (Hannover, Germany), Ericsson (Stockholm, Sweden), Nissan (Yokohama, Japan), NTT DOCOMO (Tokyo, Japan), OKI (Tokyo, Japan) and Qualcomm Technologies, but, to the best of our knowledge, no other communications have been done after that [57].

However, given the still limited number of equipped vehicles, with which cars can they actually talk to? The low penetration and the absence of a road infrastructure are the limit of equipped cars today and managing the transition toward a full penetration is not simple. Mandatory rules and suitable business models could represent a real motivation for manufacturers to move forward in an efficient way, equipping a critical mass of vehicles that otherwise would take many years to develop.

## 6. Performance Comparison of IEEE 802.11p and LTE-V2V

**Figure 3:**
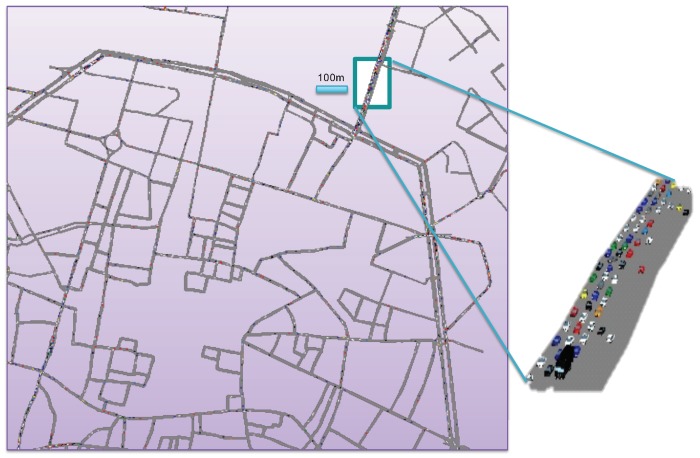
Simulated scenario: an area of 2.88 km${}^{2}$ of Bologna (Italy) downtown with 455 vehicles on average.

In this section, we present some example results to compare the performance of the two main candidates, IEEE 802.11p and LTE-V2V. To provide example results in realistic conditions, we consider the road network layout of downtown Bologna; specifically, a portion of 1.8 × 1.6 km${}^{2}$ has been simulated, corresponding to an area of 2.88 km${}^{2}$, as shown in Figure 3. Traffic traces have been generated offline with a VISSIM micro-mobility simulator and given as an input to LTEV2Vsim [58]. An average number of 455 vehicles travel in the scenario and periodically exchange beacon messages of 300 bytes with a frequency of 10 Hz.

As a performance indicator, we use the packet reception ratio (PRR), which is the ratio between the total number of successfully received messages and the total number of neighbors of each vehicle, where we consider neighbors of all those vehicles that have an Euclidean distance from the source below the given awareness range (i.e., the given distance).

Concerning IEEE 802.11p, we evaluated the performance of MCS 3, which corresponds to the use of a quadrature phase shift keying (QPSK) modulation and a coding rate of 1/2 (i.e., 6 Mbps), suggested as the best compromise between range and duty cycle by [59] and, for comparison, MCSs 1 and 8, since MCS 1 corresponds to BPSK modulation and a coding rate of 1/2, providing the lower raw data rate (i.e., 3 Mbps), but the higher reliability and MCS 8 corresponds to 64-quadrature amplitude modulation (QAM), coding rate 3/4, providing the higher raw data rate (i.e., 27 Mbps), but the lowest coverage. MCS 3 is a somehow “average” best choice, but, if we assume a sparse network, the best choice would be to use MCS 1, which allows higher range, since the channel occupation would not be a problem; in the case of a road congestion, instead, maybe using MCS 8 could be a better trade-off for the range (which would not be a problem, given that cars move slowly and close to each other) with shorter transmissions over the wireless channel. Hence, we consider various MCS options.

For the LTE-V2V system, we assume Sidelink Mode 4 (i.e., autonomous resource allocation) and different MCSs: MCS 4 and 7, corresponding to QPSK modulation and MCS 12 and 20, corresponding to 16QAM modulation. Similar considerations as those for IEEE 802.11p could also be applied for the choice of MCSs in LTE (further details on MSC and beacon resource (BR) allocation for LTE-V2V Mode 4 can be found in [5].).

For the channel model, we refer, for both IEEE 802.11p and LTE-V2V, to the one proposed by 3GPP, the Winner+ model B1, with shadowing log-normally distributed and having standard deviation of 3 dB in line of sight (LOS) and 4 dB in non line of sight (NLOS) [60]. The main settings are summarized in Table 3.

**Figure 4:**
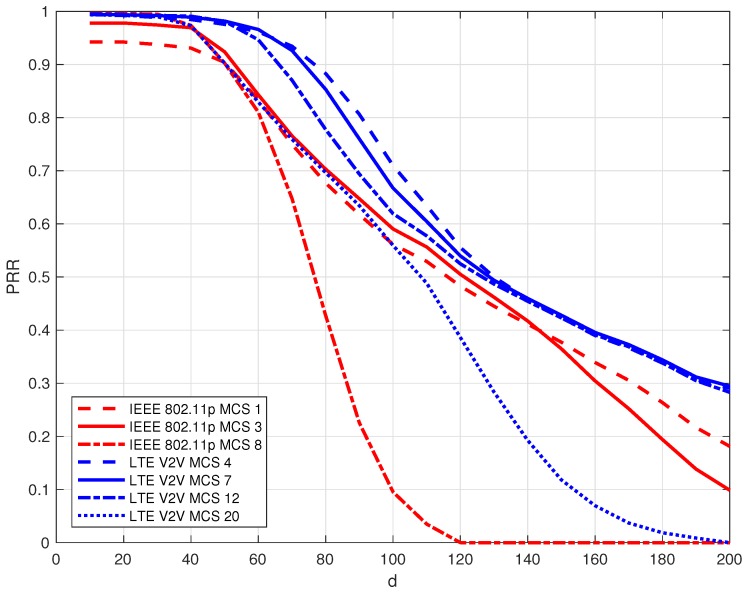
Packet reception ratio (PRR) vs. transmitter–receiver distance: comparison between IEEE 802.11p and LTE-V2V for different MCSs.

In Figure 4, we plotted the PRR as a function of the transmitter–receiver distance for IEEE 802.11p and LTE-V2V varying the MCS. As expected, the curves are monotonically decreasing as the distance increases. It can be observed that the performance is highly affected by the adopted MCS and it is not easy a priori to predict which technology outperforms the other. For distances lower than 50 m, both of the technologies provide a PRR close to 1 and then LTE-V2V outperforms IEEE 802.11p when adopted with a MCS lower than 20. By adopting LTE-V2V with MCS = 20, the PRR is very similar to that of IEEE 802.11p with MCS 1 and 3 up to 100 m; then, IEEE 802.11p outperforms LTE-V2V. The worst performance, in this urban scenario, is provided by IEEE 802.11p with MCS = 8 since, as is known, it has the lower reliability in terms of covered distance; in fact, its PRR falls rapidly after 50 m of distance between the transmitter and the receiver.

## 7. Conclusions and Perspectives

In this paper, we surveyed the main pushes, requirements and enabling technologies for short-range V2V-based VSNs.

In terms of availability, IEEE 802.11p and its related standard have the desirable features of not relying on network infrastructure and being fully distributed. Although RSUs are required to enable some of the envisioned services, vehicles equipped with this technology are able to talk to each other directly, even where there is no infrastructure deployed. On the other hand, the uncoordinated channel access strategy used by IEEE 802.11p is unable to fulfill the (deterministic) latency, reliability and capacity requirements of future V2X use cases toward autonomous vehicles. Perhaps LTE-V2X will gain momentum and open the road to 5G vehicular networks, where the best of both worlds will be combined together and with other technologies. The performance of the two technologies are highly dependent on distance and settings, such as the MCS and the resource allocation algorithm.

Which technology will be first integrated on board will also depend on international rules and business models. Only a mandate can, in fact, support manufacturers to develop a critical mass of equipped vehicles. In this context, the US Department of Transportation is for mandated deployment of V2V communications for safety applications based on IEEE 802.11p. The EU promotes C-ITS implementation, but has not given precise rules in the direction of an implementation in the short term.

Beyond Day 1 use cases, C-ITS and Automation applications are investigated and developed and the European Commission installed new projects on research, innovation and deployment to stimulate the further growth of new C-ITS and automation use cases making use of hybrid communication architectures to increase the effect on the European safety objectives. The industry is also looking at technology enhancement, improved quality of service and extended services.

The 5GAA and the European Association for Transactional Analysis (EATA) are active in developing C-ITS services and realize the first implementations of the LTE-V2X technologies. The EATA focuses on hybrid communications including ITS-G5 for short-range communication. The 5GAA so far has five vehicle manufacturer members and focus on C-ITS and Automated applications. They do not reference any specific C-ITS application standards, and application titles do not correspond to those used in these C-ITS standards identified as Day 1 applications defined by the EC C-ITS Deployment Platform.

In the long-term, connectivity will also have to converge with automation in order to achieve cooperative, connected and automated mobility (CCAM) and provide the most benefits in terms of road safety and efficiency for future connected cities, thus asking for even more challenging requirements and use cases, which will have to be addressed in the (not so) long-term.

## Figures and Tables

**Table 1:** Example requirements for typical V2V-related applications.

Application	Communication Type Rate (Hz)	Update	End-To-End Latency (ms)	Data Rate (kb/s)	Positioning (cm)
Emergency electronic brake lights	Periodic broadcast	1–10	100	1–10	-
Pre-crash sensing		10	20	20–25,000	<50–100
Pre-collision brake assist		50	50	20–25,000	30
Emergency vehicle warning		10	100	≥10	-
Overtaking vehicle warning		2–10	100	10–5000	30
Lane change assistance		2–10	100	10–5000	30
Cooperative glare reduction		2	100	≥10	-
Merging traffic turn collision risk warning		2–10	100	10–5000	-
Cooperative collision warning		10	100	≥10	30
Cooperative navigation		1–10	100	10–2000	<100
Adaptive cruise control		1–10	100	10–2000	<100
Highway platooning		≥2	≤10	≥10	30
Emergency or slow vehicle warning	Event-driven	10	100	≥10	-
Wrong way driving warning		≥1 & ≤10	100	1–10	<100
Stationary vehicle warning		≥1 & ≤10	100	1–10	<500
Traffic condition warning		1–10	100	1–10	<500
Intersection warning		10	100	10	<100
Post-crash warning		10	100	≥10	<100
Cooperative adaptive cruise control		1–10	100–300	10–2000	30

**Table 2:** Channel allocation for ITS-G5 in Europe and IEEE 802.11p in the US.

G5 Channel	IEEE 802.11p Channel	Center Frequency (GHz)	G5 TX Power Limit EIRP (dBm)	IEEE TX Power Limit EIRP (dBm)
G5-CCH	180 SCH	5.9	33	23
G5-SCH2	178 CCH	5.89	23	44.8
G5-SCH1	176 SCH	5.88	33	33
G5-SCH3	174 SCH	5.87	23	33
G5-SCH4	172 SCH	5.86	0	33
G5-SCH5	182 SCH	5.85	0	23
G5-SCH6	184 SCH	5.91	0	40

**Table 3:** The main simulation parameters and settings.

**Common Parameters (Symbol)**	**Value**
Beacon frequency	10 Hz
Beacon size	300 bytes
Channel bandwidth	10 MHz
Equivalent radiated power	23 dBm
Antenna gain at the receiver	3 dB
Path loss at 1 m	47.86 dB
Loss exponent	2.20
**IEEE 802.11p**	**Value**
MCS	1, 3 and 8
Carrier sensing sensitivity	−85 dBm
Noise power	−95 dBm
**LTE-V2V**	**Value**
Noise power over an RB	−110 dBm
MCS	4, 7, 12 and 20
